# Precision oncology using ex vivo technology: a step towards individualised cancer care?

**DOI:** 10.1017/erm.2022.32

**Published:** 2022-10-03

**Authors:** Sophie T. Williams, Greg Wells, Samantha Conroy, Hannah Gagg, Richard Allen, Ola Rominiyi, Thomas Helleday, Katie Hullock, Catherine E. W. Pennington, Juha Rantala, Spencer J. Collis, Sarah J. Danson

**Affiliations:** 1 Department of Oncology & Metabolism, The University of Sheffield Medical School, Sheffield S10 2SJ, UK; 2 Sheffield Teaching Hospitals NHS Foundation Trust, Sheffield, UK; 3 Academic Unit of Urology, The University of Sheffield Medical School, Sheffield S10 2RX, UK; 4 Helleday Laboratory, Karolinska Institutet, Solnavägen 1, 171 77 Solna, Sweden; 5 Sheffield Experimental Cancer Medicine Centre, Sheffield Teaching Hospitals NHS Foundation Trust & University of Sheffield, Sheffield S10 2SJ, UK; 6 Misvik Biology Ltd, Karjakatu 35 B, FI-20520 Turku, Finland; 7 Sheffield Institute for Nucleic Acids (SInFoNiA), University of Sheffield, Sheffield S10 2TN, UK

**Keywords:** Cancer, drug screening, ex vivo, functional precision medicine, personalised medicine, targeted therapy

## Abstract

Despite advances in cancer genomics and the increased use of genomic medicine, metastatic cancer is still mostly an incurable and fatal disease. With diminishing returns from traditional drug discovery strategies, and high clinical failure rates, more emphasis is being placed on alternative drug discovery platforms, such as ex vivo approaches. Ex vivo approaches aim to embed biological relevance and inter-patient variability at an earlier stage of drug discovery, and to offer more precise treatment stratification for patients. However, these techniques also have a high potential to offer personalised therapies to patients, complementing and enhancing genomic medicine. Although an array of approaches are available to researchers, only a minority of techniques have made it through to direct patient treatment within robust clinical trials. Within this review, we discuss the current challenges to ex vivo approaches within clinical practice and summarise the contemporary literature which has directed patient treatment. Finally, we map out how ex vivo approaches could transition from a small-scale, predominantly research based technology to a robust and validated predictive tool. In future, these pre-clinical approaches may be integrated into clinical cancer pathways to assist in the personalisation of therapy choices and to hopefully improve patient experiences and outcomes.

## Introduction

Despite advances in cancer genomics and the increased use of genomic medicine, metastatic cancer is still mostly an incurable and fatal disease. Cancer often develops resistance, despite a good initial response to therapies, and treatment based on genetic diagnosis is not always effective (Ref. 1). Sequencing of the cancer genome alone is not enough to elucidate phenotypic properties of cancer cells as many other stages of biology need to be factored in, for example, gene expression and translation, epigenetic and post-transcriptional changes, microenvironment dynamics and the balance between the body's immune system and the cancer. Simultaneous consideration of these factors is challenging, and methods for understanding the interplay between them deficient.

The delivery of novel drugs into clinical practice is dependent upon robust preclinical models. The lack of drug efficacy (when a drug is tested in the clinic) is one of the leading causes of drug development failure (Ref. 2). Preclinical research has traditionally focused on two main approaches, in vitro and in vivo, testing target specificity in vitro, efficacy in an in vivo model (often murine models in cancer drug development) and response in early to late phase human clinical trials. However, with diminishing returns, and high clinical failure rates, more emphasis is being placed on alternative drug discovery routes, such as ex vivo approaches.

For the purpose of this review, we will define ex vivo approaches as any method of conducting a drug screen directly on primary viable cancerous cells, or solid tumour tissue taken directly from a patient. The term ex vivo describes a variety of techniques, such as two-dimensional (2D) or three-dimensional (3D) cell cultures, patient-derived explants (PDEs) or xenograft models, which are utilised to capture the complexity of a disease outside of the human body. They aim to bring biological relevance and inter-patient and intra-patient variability to an earlier stage of drug discovery, and to offer more precise treatment stratification for patients (Refs 3, 4).

**Fig. 1.**
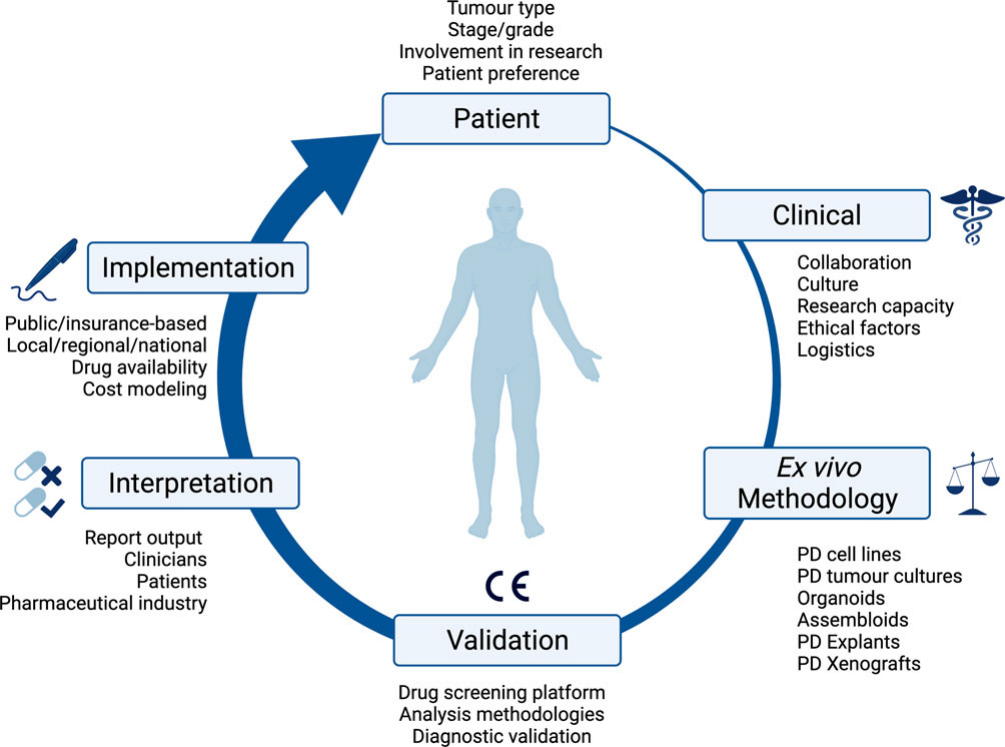
Overview of the consideration for implementing ex vivo approaches in clinical practice. PD, patient-derived; CE, Conformité Européenne.

An array of ex vivo approaches is available to researchers (see Fig. 1), and while established within the research field, only a minority of techniques have so far made it through to direct patient treatment within robust clinical trials. Encouragingly though, in these few trials, ex vivo approaches have shown very promising results, demonstrating there are possibilities when standard care options have been exhausted. This review will focus on ex vivo screening using small molecule therapeutics, but we recognise that future ex vivo techniques will need to address the biological complexity of immunotherapy agents.

## Challenges for ex vivo in clinical practice

In the era of precision medicine, personalised therapy is often equated with genomic biomarkers/profiles (Ref. 5). This has led to some highly successful therapies being directed through genetic biomarkers in specific cancers such as epidermal Growth Factor Receptor (EGFR) inhibitors in non-small cell lung cancer (NSCLC) and B-Raf Proto-Oncogene, Serine/Threonine Kinase (BRAF) inhibitors in melanoma (Refs 6, 7). However, a number of studies have shown that most patients with cancer who receive genomic testing alone do not benefit from a genomic precision medicine strategy (Refs 8, 9, 10, 11). In addition, a randomised control trial (RCT) comparing outcomes of patients matched to an off-label molecularly targeted therapy outside of its indication, versus standard treatment of care, showed no statistically significant benefit. Instead, off-label therapies were associated with greater toxicity (Ref. 12).

One strategy to improve the clinical utility of personalised therapy is to integrate functional phenotypic screening into clinical practice: determining tumour drug response ex vivo, either in isolation or in combination with genetic information. Similar types of phenotypic screening have been widespread in other areas of medicine for nearly a century and continue to represent a key area of research in these fields, for instance, testing antibiotic or antifungal effectiveness, or in predicting tuberculosis responses to therapies (Ref. 13). Although methodologically feasible, ex vivo approaches are themselves associated with several challenges in clinical application. These range from patient-, disease- and assay factors to implementation within clinical systems, which involves numerous stakeholders with different needs. Ultimately, as a technology, the main aim of ex vivo approaches is to improve patient outcomes. This review outlines the benefits of ex vivo approaches, as well as the challenges to be overcome to meet this overarching aim.

### Patient recruitment and sample acquisition

**Fig. 2.**
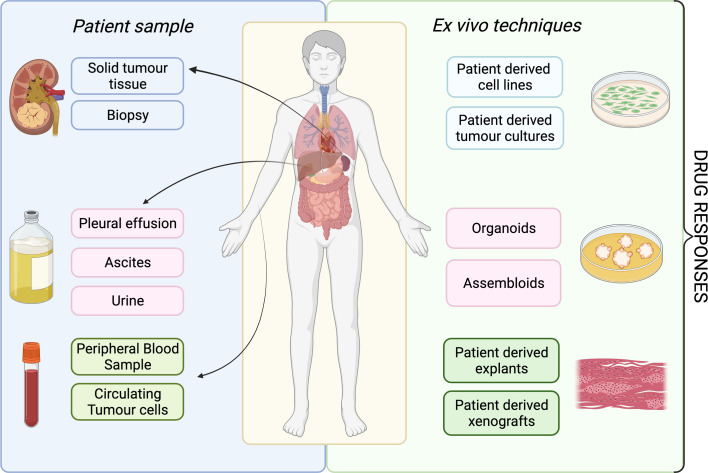
Overview of available ex vivo technologies and potential patient samples.

A patient's tumour type, stage and treatment course are major factors to consider in delivering ex vivo approaches into clinical practice. The development of ex vivo approaches initially accelerated in haematological malignancies, as malignant cells can be readily acquired from a minimally invasive blood sample (Fig. 2). This process has been much slower for solid cancers, partly because sample acquisition in most of the cases involves an invasive biopsy or surgical resection, and considerations have to be made regarding sufficient tissue for parallel histological diagnosis. It may be more attractive to propose using effusions, ascites, cerebral spinal fluid and peripheral blood derived circulating tumour cells to probe the tumour cell populations in solid malignancies, given that it is easier to access. All the common solid tumour sampling techniques (biopsy, surgical resection, effusions drainages or peripheral blood draw) are associated with sampling bias. This occurs through underreporting the heterogeneity of the entire tumour, only removing surgically accessible parts, or selection bias for cells which over or underrepresent certain features of the tumour. For example, in some cancers, sampling from the invasive front or the base of a localised tumour may disrupt the histological diagnosis, and thus, these regions cannot ethically be used for research purposes. Consequently, biological features and treatment resistance profiles of locally invasive disease may be under-emphasised. Ex vivo approaches are therefore limited in providing the full scale of heterogeneity in the patient, with the defining factor being the sampling itself.

As ex vivo approaches are primarily used as a research tool at present, the implementation into clinical practice will be dependent upon the design, funding, patient recruitment of and, ultimately, the results of clinical trials (Fig. 1). The type of tissue available intersects with the availability of patients to access clinical trials, and therefore impacts the validation of ex vivo approaches in clinical practice. Cancers associated with effusions or ascites are inherently at a more advanced stage, and are associated with reduced quality of life, performance status and worse overall survival. These factors influence the recruitment of patients to clinical trials: patients need to be fit enough to tolerate possible treatment side effects, and this becomes less likely for those with more advanced disease. It is also important to recognise that access to clinical trials is not uniform across the UK, and trial participants do not always represent the general population because of geographical, economic and demographic factors.

### Clinical pipeline

Implementing an ex vivo screening platform is a multidisciplinary project. There must be collaboration between a number of stakeholders across the patient's treatment journey (Fig. 1). Once a patient is referred for investigation of a potential cancer or recurrence, there is initial discussion within the multidisciplinary team (MDT) between nurse specialists, oncologists, radiologists, surgeons, clinical scientists and histopathologists about the best approach for the patients cancer, whether that be for diagnostic biopsy, surgical resection, neoadjuvant, adjuvant or palliative chemotherapy or radiotherapy, or best supportive management. Once a potential patient with the disease of interest, and on a clinical treatment path which aligns with ex vivo screening has been identified, it is then the responsibility of the research or clinical team, to identify, contact and discuss ex vivo testing with the patient. If the patient consents to participate, there is then coordination with the surgical team about the resection specimen, how it is handled (e.g. use of fresh tissue and avoidance of formalin) and directed both to histopathology and the ex vivo laboratory. Once the sample has been processed through an appropriate ex vivo pipeline, the resulting data then must be communicated in a time frame that is practical to direct patient care, and in a manner that is intuitive to the clinical team. There are many routes to achieving this overall pipeline, which are likely to reflect the institutional research culture, and in the initial validation stages, collaboration between clinicians, surgeons and researchers. The next challenge would then be access to potentially off-label repurposed treatments and how these are funded; this would differ between healthcare system and countries. Examples in this review include application within Finland (Refs 14, 15, 16, 17, 18), Austria (Refs 19, 20, 21), Australia (Ref. 22), USA (Ref. 23) and South Korea (Refs 24, 25).

### Sample handling and stability

The last decade has seen a paradigm shift in the collection of diagnostic tissue. Prior to this tissue was rapidly fixed in formalin, often in theatre, before reaching a pathologist. Recently, the introduction of genetic analysis in the form of next-generation sequencing (NGS) has made the collection of fresh tissue, which is rapidly frozen as common practice, without the use of formalin. This means protocols and safe operating procedures are already in place for the transfer of fresh tissue to pathology for dissection, tumour sampling and flash freezing for genetic analysis. These protocols could be readily optimised and implemented for the collection or storage of live fresh tissue for ex vivo screening. However, sample stability and viability from resection to ex vivo processing needs to be established and validated, this will be influenced by the transfer method used. Transfer possibilities include cryopreserving solid tissue, on ice or refrigerated at 2–8°C, or in media/saline for shorter term transfer times. Cryopreservation has been assessed in haematological cancers using 2D screening techniques (Ref. 26) and solid tumours using explants (Ref. 22). However, this area will require clinical validation prior to ex vivo screening being used to direct patient treatments. Sample stability will also inform whether ex vivo analyses need to be undertaken at a local site, or whether a central regional or national ex vivo processing laboratory could be established.

## Establishing clinical effectiveness: examples from haematological malignancies

Haematological malignancies were the first cancers to demonstrate the clinical benefit of ex vivo approaches. An important early clinical trial of ex vivo-directed therapy determined the sensitivity of acute myeloid leukaemia (AML) blast or healthy-control mononuclear cells to a number of therapeutic drug compounds, and for a small cohort, fed this information back to a treating clinician to deliver ex vivo-directed therapy (Ref. 27). In all, eight patients were treated with ex vivo determined therapies, and seven demonstrated disease control, three with disease response meeting LeukaemiaNet criteria. Importantly, this study took several novel approaches not yet widely adopted in other studies at that time (Refs 28, 29, 30) working in diseases with relatively accessible patient samples (i.e. peripheral blood samples), and low genomic alteration rate and selecting cancer-specific cell populations, rather than measuring total culture viability. Some of these haematological-cancer specific factors may have contributed to the markedly higher estimated clinical approval success rate for compounds studied in haematological indications, compared with those studied in solid tumours (Ref. 30). These methods continue to demonstrate clinical potential: in a pilot clinical trial of 12 patients with refractory AML, three (Ref. 4) responded to treatment guided by ex vivo drug sensitivity testing, although the study was not randomised, and the control arm, receiving non-ex vivo therapy were not well matched (Ref. 31) (see later section on ex vivo screening in clinical practice). In another study of 186 AML patients, ex vivo-directed treatment for 37 relapsed or refractory patients showed a 59% objective response rate (Ref. 32).

More sophisticated methods of distinguishing cancer cell populations are now available. In a recent clinical trial of ex vivo determined therapies, image-based screening (‘pharmacoscopy’) was used to distinguish target cell populations among a diverse group of patients with advanced haematological malignancies. The prospective study included 17 patients with pharmacoscopy-guided treatment: 88% (15/17) patients in this arm achieved an overall response, compared with 24% (4/17) of patients in their last treatment, and 38% (5/13) patients in a group receiving physician-decided salvage treatment. No pharmacoscopy-treated patient demonstrated progressive disease, compared with seven during the last treatment and demonstrated improved median progression-free survival (22.6 weeks) compared with a median of 5.7 weeks in the same patients with the most recent regimen (Ref. 21). A further publication from this same consortium, detailing results from an expanded clinical cohort of 56 patients, demonstrated a clinical benefit of 1.3-fold increased progression-free survival in 30 patients (54%), compared with the same-patient prior therapy (Ref. 19).

Ex vivo approaches have been utilised to explore aspects of leukaemia biology beyond drug response. Studying mechanisms of ex vivo resistance has led to greater understanding of clonal evolution in chronic myeloid leukaemia at different disease stages, and provided new insights into therapeutic susceptibility (Refs 33, 34). Ex vivo approaches are also attractive to those researching therapeutics for rare conditions, where RCTs are difficult to undertake. For example, in T-cell prolymphocytic leukaemia, in a small number of patients, ex vivo screening identified novel therapies, and outperformed molecular characterisation alone in correlating with clinical response to treatment (Ref. 35). The success of ex vivo-based techniques used to direct patient treatment in haematological cancers enabling sensitive quantification of cancer cell populations holds promises for similar methodologies to be used in solid cancers.

## Which methodology to choose in solid tumours?

The initial studies describing use of ex vivo chemosensitivity testing for assessment of therapy efficacy in solid tumours date back to the late 1970s (Refs 36, 37). Since then, a plethora of ex vivo platforms have been reported which attempt to capture the complexity of a disease outside of the human body: whether this be in 2D or 3D cell models, PDEs or xenograft models. However, in short, there is no perfect model which fully represents the complex dynamics of drug tumour interaction observed in patients. In this section we discuss the most clinically relevant models and their advantages and disadvantages for implementation within a clinical setting.

### Patient-derived cell lines

Patient-derived cell lines (PDCLs) describe the generation of cell lines derived from the primary tumour tissue using cell dissociation and passaging in define media conditions to propagate a specific cell type. They reflect the ‘in vitro’ end of the ex vivo approaches spectrum. PDCLs provide a midway point between patient-derived cultures, described in section ‘Patient-derived cultures’, and established commercial cancer cell lines.

There are multiple examples of successful ex vivo screening studies using PDCLs. In human papillomavirus -negative head and neck squamous cell carcinoma, Lepikhova *et al*. generated 45 primary cell lines from primary or recurrent tumour tissue samples (Ref. 38). Screening with 220 compounds identified selective sensitivity to EGFR, Mitogen-Activated Protein Kinase Kinase 1 (MEK) and Mechanistic Target Of Rapamycin Kinase (mTOR) inhibitors across eight phenotypic subgroups. Although EGFR and MEK mutational status of the cell lines did not correlate with respective ex vivo drug sensitivity, several other candidate genes involved in membrane trafficking and transport were identified that did correlate with drug sensitivity. In glioblastoma (GBM), Stringer *et al*. developed 12 patient-derived cell lines and compared targeted exome sequencing of the parental tumours, patient-derived cell lines at 8–15 passages and patient-derived cell line xenografts in non-obese diabetic/severe combined immunodeficient mice (Ref. 39). Of the 12 primary cell lines generated, five displayed the same gene expression signature GBM subtype as their parental tumours ex vivo; however, this increased to 7/12 within the xenografted primary cell line tumours, suggesting the derived lines did diverge faster than the xenografted model. In one exemplar study, Kim *et al*. utilised PDCLs in a prospective observational study looking at NSCLC (Ref. 40). The authors generated 23 PDCLs from 96 malignant effusions (24%). Of these, nine PDCLs had known *EGFR* drivers, matching the patient's genotype. All nine patients had previously been treated with EGFR TKI, prior to generation of the PDCL. Of these, five of the patients had been treated with a first-generation EGFR TKI and were subsequently treated with osimertinib, a third-generation EGFR TKI. The PDCLs were also screened with osimertinib and demonstrated a correlation with the patient's clinical outcome. Kim *et al*. suggested this predictive model could be used to direct patient treatment. This approach has also been adopted in a case series of related, rare, relapsed sarcoma cancers (Ref. 41).

Conditional reprogramming is an alternative method used to rapidly expand primary patient tumour (and normal) cell populations from tumour samples. Kettunen *et al*. explored ex vivo drug screening in bladder cancer by generating four conditionally reprogrammed cell (CRC) lines (Ref. 42). Only 2/4 (50%) CRCs maintained mutational consistency to their parental primary tumours, reflecting subpopulation overgrowth – in this case, of normal epithelial cells that did not harbour the mutations seen in the parental tumours. Nonetheless, their subsequent high-throughput screening did identify several standard, novel and repurposed agents. In particular, one of the more aggressive small cell bladder tumours was particularly sensitive to statins, a cheap, readily available, and safe Food and Drug Administration (FDA)-approved treatment that could be immediately added to standard of care (Ref. 42). Similar reprogramming approaches have been successfully adopted in NSCLC and pancreatic ductal adenocarcinoma (Refs 24, 43).

PDCLs can easily be assayed in a high-throughput manner, where monocultures can grow over extended incubation periods, are less technically challenging to assay and require less computational complexity to analyse as CellTitre-Glo^®^ (CTG) and 2,5-diphenyl-2*H*-tetrazolium bromide (MTT) can be utilised. However, they are also limited by their simplicity: the plasticity of culturing cells in vitro can result in divergence from their parental tumours with increasing passage number. These models also loose the tumour microenvironment removing the possibility to assay the multiple cell types originally present within the tumour. The successful generation of PDCLs is also low at 24, 58 and 66% in NSCLC, sarcoma and bladder cancer, respectively.

### Patient-derived cultures

Patient-derived cultures describe the generation of a cellular suspension which retains multiple cell populations. The cellular suspension is not passaged prior to ex vivo drug screening. The cellular suspension can be derived from the dissociation of a solid tumour, or extraction of cellular components from liquid malignant fluid (such as malignant pleural or ascitic fluid).

This approach has been utilised in several case reports of rare cancers, where efficacy of treatment options remains largely under-researched and lacks evidence because of low patient numbers and difficulty accessing funding. Three exemplar case studies have been published utilising this technique to direct patient treatment. In two cases of the rare form of gastric-originating Krukenberg tumour, which had progressed despite first-line chemotherapy and had no standard of care second-line options available, an ex vivo platform identified the tyrosine kinase sunitinib as effective. Impressively, in one of the patients who was fit enough for second-line therapy, sunitinib resulted in stable disease for 5 months (Ref. 15). A similar approach was used for a patient with a rare, metastatic epithelial-myoepithelial salivary gland tumour, with no further standard treatment options available. Ex vivo screening identified effective novel therapies and also differential sensitivities to treatment between the myoepithelial and epithelial cell lineages (Ref. 17). Subsequent treatment with the mTOR inhibitor everolimus reduced the patient's metastatic burden by 25%, an effect which was sustained for a period of 11 months. Finally, in a single case of recurrent thymoma, presenting with multiple mediastinal masses 11 years after initial diagnosis, surgically resected samples were analysed using an ex vivo platform. Multiple EGFR-targeting therapies were identified among the CK19-staining population (an epithelial marker associated with thymoma). Cetuximab, an anti-EGFR therapy, was given to the patient, and resulted in stable disease for 13 months (Ref. 14). It is interesting to note that while this drug class was used in the drug screen, cetuximab itself was not in the drug screen, thus highlighting the potential of ex vivo approaches to use a range of experimental and approved compounds to identify approved drug classes that might confer tailored therapeutic benefit. Table 1 highlights studies in which ex vivo approaches have been used to direct patient therapy.

**Table 1.** Summary of ex vivo models that have been utilised to direct patient treatment

Author, year	Cancer type	Primary clinical outcome	Patients recruited (*n*)	Samples type	Models generated (*n*, %)	Primary outcome measure met (*n*, %)	Number of drugs screened
Arjonen *et al*. (Ref. 14)	Krukenberg tumours	To use ex vivo PDC sensitivities to prospectively inform patient treatment	2	2 needle biopsies, 2 malignant ascites	2 models (100%)	1 patient (50%)	120 drugs screened. Patient treated with sunitinib then trastuzumab
Mäkelä *et al*. (Refs 16, 17)	Salivary gland tumour	To use ex vivo PDC sensitivities to prospectively inform patient treatment	1	2 core biopsies	1 PDC (100%)	1 patient (100%)	134 drugs screened. Patient treated with everolimus, then trastuzumab and lapatinib
Arjonen *et al*. (Ref. 15)	Recurrent thymoma	To use ex vivo PDC sensitivities to prospectively inform patient treatment	1	Surgical resection biopsy	1 PDC (100%)	1 patient (100%)	147 drugs screened. Patient treated with cetuximab
Narasimhan *et al*. (Ref. 44)	Metastatic colorectal cancer	To use ex vivo organoid sensitivities to prospectively inform patient treatment	28	Surgical resection or laparoscopy biopsy	19 models (68%)	2 patients (10%) had partial response	87 screened in 5 cases, then smaller panel 35 drugs in remaining models

PDC, patient-derived cell line.

It is also possible to undertake high-throughput ex vivo screening in 3D of patient-derived cultures using a scaffold/matrix. In a metastatic urachal adenocarcinoma that had progressed despite two lines of therapy, a 2D ex vivo model was compared with a 3D high-throughput screen. The 3D assay was longer (7 day incubation), and showed some variation in drug efficacies; most notably, growth inhibition was less potent within the 3D assay (Ref. 16). However, such differences may better reflect responses in vivo, so further development of 3D-based ex vivo platforms might be beneficial for certain tumour types (see the next section for further information on 3D-based ex vivo models).

The 2D techniques used in the case studies can simultaneously screen large numbers of drugs and yield fast results (3–5 day drug incubation time), with the potential to deliver results in a clinically relevant window (Refs 14, 15, 17). The image analysis enabled differentiation of the multiple cell types via immunofluorescence to segment cell populations, providing more targeted dose response for each population. However, the limitations include the loss of spatial tissue microenvironment and architecture, which may affect drug sensitivity analysis and reduce the efficacy of testing anti-angiogenic therapies or immunotherapies. Another key limitation is that not all solid cancers are adherent without prior passage. Although a potentially fast technique, certain chemotherapeutics require several cell divisions to impart their full cytotoxic activity, and so may demonstrate a falsely suppressed response.

### Patient-derived organoids

Organoids are miniaturised, multicellular 3D cultures which closely mimic the originating in vivo organ. Organoids are established from stem cells, including tissue specific adult stem cells (ASCs) or through the differentiation of pluripotent stem cells (PSCs) such as embryonic stem cells or induced PSCs (iPSC). In ex vivo platforms, ASCs can be obtained in the same way as patient-derived cell models. Organoids are an enticing ex vivo technology, offering the promise of greater tumoural heterogeneity, and their application in high-throughput drug screens by the generation of organoid libraries. Many excellent reviews already exist detailing the development of organoids as model systems for human physiology and drug screening (Refs 45, 46). Here we focus on publications of primary research in which organoid drug screening results have directed patient treatment.

Numerous studies have utilised organoids within an ex vivo drug screen (Refs 47, 48, 49, 50, 51, 52, 53, 54, 55, 56), including examples which test thousands of potential treatments (Ref. 57) and matched genotyping, in some cases identifying active compounds in the absence of an apparent genetic biomarker (Ref. 55). In one exemplar study, Narasimhan *et al*. implemented drug screening of an organoid biobank generated from colorectal peritoneal metastases to direct treatment for two patients, one of whom showed a partial response, despite progression on the previous line round of standard care chemotherapy (Ref. 44).

Organoids harbour the potential to more accurately mimic human 3D tissue architecture compared with 2D cell-based models, while also supporting both healthy cells and tumour cells over an extended period. Organoids can represent features of anatomical variation and tumour heterogeneity seen in primary tumour and metastases. They can reconstruct distinct brain regions, such as the hippocampus, retina and different cortical domains (Ref. 58) which are not comparable in rodents (Ref. 59). In GBM, multiple cell types persisted within organoids over a 48-week period (Ref. 60). Whole-exome sequencing at 2 weeks confirmed most somatic variants were consistent between parental tumours and corresponding organoid (Ref. 60). However, organoids can also be deficient in key cell types, including endothelial and microglial cells in CNS organoids, although some methods have been derived to address this (Refs 61, 62).

Organoids lack a blood supply and so they are limited in the size they can grow. As with in vivo solid tumours, the centres can become necrotic because of lack of oxygen and nutrients (Ref. 63). This issue may be circumvented through advances in the derivation methods (Ref. 60) or culturing methods, including co-culturing with endothelial (angioblasts) and mesenchymal precursors (Ref. 64).

There are several considerations around the derivation and properties of organoid culture models. Organoid generation requires tissue-specific niche factors to drive stem cell self-renewal, adapting a basic medium developed initially for the generation of gastrointestinal organoids (Refs 65, 66). The requirement of oncogenes to maintain ASC from biopsies can result in unwanted genetic alterations, necessitating additional experimental controls. Earlier organoid generation protocols required relatively large sample amounts, although fine needle aspiration biopsies can now be used to generate organoids, achieving between 54 and 100% success, depending upon tumour type (Ref. 67). Additional methods have been developed to best utilise small tissue amounts, such as the encapsulation microfluidics perturbation system, in combination with in silico modelling (Ref. 68). When considering the possibility of utilising patient-derived organoids for drug sensitivity screening, organoids are faster to culture than patient-derived xenografts (PDXs), but slower than patient-derived cultures. In lung cancer, establishment of organoids took approximately 4 weeks compared with 2–4 months for PDX models (Ref. 25). Success rates of organoid formation varies between cancer type and range from 100% reported in primary colon (Refs 55, 57, 65, 69), liver (Ref. 48), endometrium (Refs 49, 70, 71) and thyroid (Ref. 67), down to 16–18% in prostate (Refs 53, 72). Success can also differ between genetic subgroups: in GBM, IDH1 mutant tumours exhibited the lowest organoid generation success (67.7%) in comparison with wild-type counterparts (96.4%) (Ref. 60).

The generation of organoids can be labour intensive, especially when more complex organoids are involved. The greater the complexity of the organoid, the more variation within organoids derived from the same patient. Any automated drug screening platforms and analyses techniques must take this into account. Although iPSC cell lines can be easily generated from human cells it is often difficult to isolate the specific ASC populations responsible for disease progression. Using clustered regularly interspaced short palindromic repeats/Cas9 (CRISPR/Cas9) genome editing technology, specific mutations can be introduced, opening up new opportunities to recreate individual patients' disease and correlate drug sensitivity in vitro in a personalised fashion. This could result in the creation of organoid biobanks covering the spectrum of the cancer's mutations.

### Organoids on chips

The ability for organoids to form within a perfusion system has enabled their development into chip-based technologies. This has accelerated the development of chips to study normal physiology (Ref. 73), including the blood–brain barrier (Ref. 74), liver (Ref. 75), vascularised and perfused organs (Ref. 76), adult kidney (Ref. 77) and heart (Ref. 78), to name a few, as well as cancer disease processes, including lung (Refs 79, 80), GBM (Refs 81, 82), liver (Refs 83, 84), colorectal (Ref. 85), breast (Refs 86, 87) and pancreatic cancer (Refs 88, 89).

Chip-based technology has also been utilised to study interactions between the tumour and extracellular environment, as well as other tumour properties, such as epithelial and mesenchymal transition, angiogenesis, tumour invasion, cell migration and metastasis. The technology is particularly applicable to luminal models, where the flow of substrate/biomarkers can be detected in real time. This extends to continuous drug dosing, and its application in personalised drug screening. In one exemplar example, Mazzocchi *et al*. used a synthetic hydrogel, tumour cells from patients with mesothelioma were introduced to a microfluidic device which allowed a continuous flow of media with drug dosing, which showed a strong correlation between organoid response and clinical outcomes for the two patients tested (Ref. 80).

### Assembloids

Assembloid technologies focus on reconstituting tumour organoids, derived from patient tissue (with constituent ASCs), alongside other cellular components of the tumour microenvironment. Although organoids can reproduce many aspects of human physiology and disease, they cannot offer a defined spatial organisation of multiple cell or tissue types. Beyond this, many cell types change characteristics when they are removed from their natural environment (Ref. 46). This spatial organisation is crucial for understanding a number of cancer associated phenotypes, for instance blood–brain barrier breakdown (Ref. 90), immune-mediated interactions or tumour invasion (Ref. 91). Assembloids offer the potential of greater inclusion of extra-tumoural factors within a model, with the aim to develop a biological model which can functionally recapitulate the in vivo biology of native and tumour tissues and their interaction.

Elegant techniques developed by Birey *et al*. at Stanford University combined spheroids developed from two distinct brain regions (pallium and subpallum) to generate assembloids modelling cerebral cortical development (Ref. 92). These methods demonstrate generating functionally integrated glutamatergic and GABAergic neurons. This system showed that interneurons derived from patients with Timothy syndrome, a neurodevelopmental disorder, resulted in CaV1.2 calcium channel mutations. These patient-derived assembloids showed migrational defects, which could be rescued by manipulating L-type calcium channels. More recently Miura *et al*. derived cortico-striatal assembloids from patients with Phelan–McDermid syndrome, a neurodevelopmental disorder caused by a deletion on chromosome 22q13.3. Using these patient-derived assembloids, they detected novel disease-associated calcium-signalling dysregulation, providing the first preclinical model of this observation (Ref. 93). Both papers describe producing the assembloids from spheroids by co-embedding them into a well containing either matrigel or hydrogel, the most challenging stage of development seems to be the initial generation of spheroids from hiPS cells (Refs 92, 93). Nevertheless, this may be circumvented in the future through improvements in commercially available STEMdiff™ and AssemBloids™ kits.

One exemplar in bladder cancer, Kim *et al*. used similar techniques to generate assembloids which contained the four major constituents of the tumour environment: stromal fibroblast, endothelial, immune and muscle (Ref. 91). When assembloids were formed with T-cell-based immune microenvironment, CD8T cells infiltrated the assembloids and induced widespread apoptosis, reducing tumour mass. The same effect was observed with neo-antigen specific T cells, suggesting this platform would support the assessment/screening of immunological therapies.

### Patient-derived explants

PDEs are fresh tissue sections taken directly from the patient's surgically resected solid tumour specimen, or from a biopsy. These models retain tissue architecture and multiple lineages of cell types from the patient tumour. This makes them an attractive model for investigating drug responses in ex vivo platforms, as the tissue retains much of the tumoural heterogeneity and microenvironment. These models can also provide information on drug uptake into the more complex tumour architecture (Ref. 94). As a result of this PDEs have played an important role in drug and biomarker discovery (Ref. 95). Various explant models have been developed for several cancers to investigate drug response, including bladder cancer (Refs 96, 97, 98), NSCLC (Ref. 94), colorectal cancer (Ref. 98), ovarian cancer (Ref. 22), pancreatic cancer (Ref. 23) and prostate cancer (Ref. 99).

A variety of PDE culture methods are available, ranging from media submersion, similar to regular cell culture, all the way to the CANScript platform, which uses the patient's autologous serum and a tumour–stromal matrix support to culture the explant (Ref. 100). Although PDEs have not been used to direct patient treatment, an exemplar study from Karekla *et al*. demonstrated response to cisplatin did correlate to patient survival in a prospective clinical follow-up. However, PDE development is still in its infancy, many of the studies reported demonstrate feasibility and proof of concept data, showing these could be used to direct patient treatment and assess both stromal and tumour components. One major drawback is tissue requirement, limiting the number of drugs which can be screened, and therefore resulting in a low-throughput drug screen compared with 2D and 3D models. Additionally, imaging using fixed sections is often at relatively low resolution, and single-cell drug response is not currently feasible.

Unlike organoids and PDXs, PDEs offer a relatively fast approach to investigate drug sensitivity. As explants do not have to undergo selection or expansion prior to analysis, they are essentially ready for ex vivo testing within minutes or hours from surgical resection. Drug exposure times in PDEs varies between methods and which drugs are being investigated, spanning 1–2 h (Refs 96, 97), or 24–72 h (Refs 22, 94). These short time frames permit results to be reported within a clinically relevant window, but may not be fully representative of certain chemotherapeutics that require several cell divisions to impart their full cytotoxic activity.

Unlike other models, which are derived from primary cells, PDEs cannot be expanded or maintained. PDEs significantly increase expression of apoptosis markers at 24 and 48 h respectively (Refs 22, 94). However, Bolenz *et al*. showed bladder cancer explants to be stable for 12 days on an absorbable gelatine matrix before seeing an increase in apoptotic markers (Ref. 98). Although most studies here use fresh tissue, Ricciardelli *et al*. demonstrated derivation of PDEs can be accomplished from cryopreserved tissue, potentially expanding the ex vivo applications of such PDEs (Ref. 22).

### Patient-derived tumour xenografts

Traditional in vivo models involve the implantation of cancer cell lines into immunodeficient mice to generate tumours. However, these models often lack complete heterogeneity of cell types, or the histology and molecular changes seen within the tumour microenvironment that they are intended to mimic (Ref. 101). PDX models, where surgically removed tumour fragments or patient-derived organoids are implanted into immunodeficient mice, offer a model that retains the histology of the original tumour, the various cell types within it and the genetic markers of the parental tumour (Refs 102, 103).

PDX models have been shown to recapitulate pharmacological responses of the parent tumours. In a clinical trial designed to assess the combination of a BRAF (dabrafenib) and MEK (trametinib) inhibitors in patients with BRAF V600-mutant metastatic colorectal cancers, Corcoran *et al*. successfully generated PDX models from four out of five patients using pre-treatment biopsies. PDX response to treatment correlated with the tumour response in the patient from which they were produced, successfully identifying resistant tumours (Ref. 104). In prostate cancer, a PDX model was established from a single patient that retained androgen sensitivity. Although no drug screening was performed on the PDX model, it was used to serially produce organoids that maintained similar physical and molecular traits of the primary tumour. Although drug screening on the PDX-derived organoids was not correlated with patient treatment and response, screening results were compared alongside two other PDX-derived organoids, which highlighted the link in responses to targeted therapies and targeted pathway analysis (Ref. 105).

PDX models termed MiniPDX have been adapted to integrate into a hollow fibre capsule enabling in vivo responses to be determined within 7 days. Either derived direct from the host or first-generation xenograft mice, tumours were resected and digested with blood and fibroblasts removed using magnetic beads before being added to the hollow fibre capsules. This resulted in the loss of the host tumour microenvironment, but allowed for the quick expansion of MiniPDX that facilitated the rapid testing of compounds. This technology is currently registered within a clinical trial (NCT03786848) (Ref. 106).

PDX ‘Avatar’ trials utilise PDX technology to follow patient response in a clinical time frame. Once a patient is enrolled as part of the clinical trial, a PDX avatar is generated using pieces of the patient's tumour. The mouse avatar is then treated simultaneously with the same treatments as the host patient to provide personalised treatment (Refs 107, 108, 109, 110). There are several interventional clinical trials being conducted using PDX avatar response to therapy to guide treatment decisions and patient care (see Table 2).

**Table 2.** Summary of current or recently published clinical trials using ex vivo methodology

Trial registration number, status	Study title	Study type	Estimated enrolment
NCT04267081	Study of venetoclax in combination with azacitidine in AML patients selected using ex vivo drug sensitivity screening (VenEx)	Interventional	100
Recruiting			
NCT04561453	Feasibility study of multi-platform profiling of resected biliary tract cancer	Observational	20
Recruiting			
NCT03739177	Onco4D™ biodynamic chemotherapy selection for bladder cancer patients	Observational	150
Unknown			
NCT03561207	3D-Predict Registry: 3D prediction of patient-specific response using ex vivo interrogation of live cells from tumours (EV3D)	Observational	570
Recruiting			
NCT03164863	Feasibility study of motility contrast tomography (MCT) for predicting therapeutic response	Observational	150
Unknown			
NCT04298489	Personalised drug sensitivity test for late stage, potentially operable gastrointestinal cancer using patient-derived primary cell culture	Interventional	40
Unknown			
NCT04470947	Comprehensive genomic profiling and next generation functional drug screening for patients with aggressive haematological malignancies (EXALT-2)	Interventional	150
Recruiting			
NCT03860376	Ex vivo drug sensitivity testing and mutation profiling	Observational	25
Active, not recruiting			
NCT03655015	Patient-derived organoid model and circulating tumour cells for treatment response of lung cancer	Observational	150
Recruiting			
NCT03979170	Patient-derived organoids of lung cancer to test drug response	Observational	50
Recruiting			
NCT03453307	The correlation study of the drug sensitivity between ex vivo model of non-small cell lung cancer (NSCLC) patient-derived organoids and clinical response in NSCLC patients	Observational	100
Unknown			
NCT04555473	Translational analysis in longitudinal series of ovarian cancer organoids	Observational	48
Recruiting			
NCT03943316	Single institution prospective laboratory study of cancer and immune cells in the ascites fluid of ovarian cancer patients to test alternative therapies	Observational	100
Recruiting			
NCT03831230	Constitution of ex vivo ovarian tumour models for the validation of the interest of innovative therapies and the search for tumour or circulating biomarkers predictive of treatment response	Observational	160
Recruiting			
NCT04736043	Development of a prediction platform for adjuvant treatment and prognosis in pancreatic cancer using ex vivo analysis of organoid culture	Observational	300
Recruiting			
NCT03821870	Predictive value of in-vitro anti-cancer therapy sensitivity testing on tumouroids from patients with metastatic pancreatic cancer	Observational	30
Recruiting			
NCT03961737	Study of the response to irradiation on prostatic explants ex vivo, as a predictive factor of the clinical response to irradiation of prostate cancers (EXPLANT)	Interventional	92
Recruiting			
NCT04349293	Ex vivo evaluation of the reactivity of the immune infiltrate of cancers to treatments with monoclonal antibodies targeting the immunomodulatory pathways	Interventional	150
Unknown			
NCT05231655	Ex vivo determined cancer therapy (EVIDENT)	Observational	600
Recruiting			
NCT02646228	Establishment of patient-derived cancer cell models to interrogate novel molecular targets in metastatic cancer	Observational	500
Recruiting			
NCT04745975	Guided treatment based on Mini-PDX in metastatic triple negative breast cancer (GUMPTION)	Interventional	100
Recruiting			
NCT03786848	Personalised Mini-PDX for metastatic CRPC	Interventional	15
Unknown			
NCT03219047	Patient-derived xenografts in personalising treatment for patients with relapsed/refractory mantle cell lymphoma (EXPLORE)	Interventional	50
Unknown			
NCT02312245	Avatar-directed chemotherapy in treating patients with ovarian, primary peritoneal, or fallopian tube cancer	Interventional	240
Recruiting			
NCT02752932	TumorGraft-guided therapy for improved outcomes in head and neck squamous cell cancer	Interventional	41
Completed			
NCT04373928	Personalised precision diagnosis and treatment of pancreatic cancer (PPDTPC)	Interventional	100
Recruiting			
NCT03263663	Optimisation of individualised therapy for CRCs with secondary RESISTance towards anti-EGFR targeted therapy using an avatar model	Observational	1000
Recruiting			
NCT02795650	Personalised therapy for metastatic ADPC determined by genetic testing and avatar model generation (AVATAR)	Interventional	146
Active, not recruiting			
NCT03133273	Study of the therapeutic response and survival of patients with metastatic colorectal cancer (stage IV) and treated according to the guidelines of a chemosensitivity test, oncogramme (ONCOGRAM)	Interventional	256
Recruiting			
NCT02927106	Beat AML core study	Observational	22
Completed			
NCT01190241	Targeted therapy selection based on tumour tissue kinase activity profiles for patients with advanced solid malignancies, an exploratory study	Interventional	45
Terminated			
NCT04450706	Functional precision oncology for metastatic breast cancer (FORESEE)	Interventional	15
Recruiting			
NCT03890614	Novel 3D haematological malignancy organoid to study disease biology and chemosensitivity (organoid)	Observational	70
Recruiting			
NCT03896958	The PIONEER initiative: precision insights on *N*-of-1 ex vivo effectiveness research based on individual tumour ownership (precision oncology)	Observational	1000
Recruiting			
NCT04768270	The culture of ovarian cancer organoids and drug screening	Observational	30
Recruiting			
NCT04755907	3D Bioprinted models for predicting chemotherapy response in colorectal cancer with/without liver metastases	Observational	120
Recruiting			
NCT04842006	Systemic neoadjuvant and adjuvant control by precision medicine in rectal cancer	Interventional	93
Recruiting			
NCT04826913	High-throughput screening device based on 3D nano-matrices and 3D tumours with functional vascularisation	Observational	100
Not yet recruiting			
NCT03336931	PRecISion medicine for children with cancer (PRISM)	Observational	550
Recruiting			
NCT04602702	Hyper-personalised medicine using patient-derived xenografts (PDXovo) for renal cell carcinoma patients	Observational	50
Recruiting			
NCT03878524	Serial measurements of molecular and architectural responses to therapy (SMMART) PRIME trial	Interventional	40
Recruiting			

AML, acute myeloid leukaemia; CRPC, castration-resistant prostate cancer; ADPC, metastatic adenocarcinoma of the pancreas; CRC, colorectal cancer.

However, there are some major limitations to PDX models. The model generation is costly, and take too long to realistically provide results in a clinically relevant time frame in many cancers. It can take 4–8 months for a model to be fully established (Refs 106, 111) and is not sufficiently tailored to identify individuals who will benefit from future PDX generation and screening upfront. Although the histology of the PDX tumours closely resembles that of the host, overtime stromal cells of the tumours are eventually replaced by murine cells, altering the paracrine signalling that can control tumour growth (Ref. 112). Immune mechanisms are inherently altered by the use of immunocompromised mice for engraftment success and tumoural evolution is known to diverge between humans and mice (Ref. 113), potentially affecting the efficacy of immunotherapy in PDX models. Humanised PDX models aim to address this, using the implantation of human CD34+ haematopoietic stem cells or the infusion of human peripheral blood which provide limited immune cell types with a short lifespan (Ref. 114). The humanised CD34 model is currently being tested for its clinical relevance in NSCLC (Ref. 115).

One overarching drawback is the reliance on animal testing, with protocols often requiring multiple animals per patient. Organisations such as the National Centre for the Replacement, Refinement and Reduction of Animals in Research (NC3Rs) are supporting alternative ex vivo technologies in order to advance pharmaco-phenotypic screening without such intense reliance on animal models.

## What should be measured?

Once an ex vivo methodology has been agreed on, the next step is to consider how to measure the response to potential treatments. The methods described in this section are all phenotypic screens: measuring an observable physical response of ex vivo approaches to dosed drug treatments.

Ex vivo approaches where the tissue retains its architecture is an advantage, such as PDE or PDX approaches as drug penetration can be measured (Ref. 94). However, proliferation and apoptosis measurements commonly rely on immunohistochemistry (IHC) marker such as Ki67 and cPARP/caspase-3, with haematoxylin and eosin staining define gross areas of tumour and normal cells, which results in lack of meaning full single-cell data (Ref. 22). This is also observed with readouts, such as CTG and MTT, which can readily be applied to PDCLs, patient-derived cultures and more complex 3D models. Although this measurement is relatively simple compared with other approaches, it lacks cell population differentials and single-cell information. However, the advantages for both of these outputs are low cost, well-established methodologies and relatively simple analysis.

More complex systems for single cell, or single-cell type approaches are necessary to add greater biological meaning to the results. A recent review by Krall *et al*. demonstrates the trade-off between scalability and information content, regarding single-cell populations (Ref. 116). There are some advantages to certain single-cell screening techniques, beyond their automation and information content. High-content microscopy can give information about cell populations at the single-cell level, but for solid tumours this comes at the cost of more complex processing protocols that robustly remove unwanted debris and red blood cells from the cultures for clean imaging (Refs 14, 15, 18, 19, 21, 26). Imaging mass cytometry is gaining more interest, although remains relatively expensive for clinical application and validated analysis methods are still in development (Ref. 117). In solid tumours, there is also the challenge of non-adherent cell types, which need to be embedded within a matrix, or fixed onto a microscopy plate to achieve accurate high-content microscopy, without introducing a reporting bias by removing a subset of unfixed cells from the final analysis. Microscopy can be adapted to image both in a 2D and 3D manner, using *z*-stacking, which is more relevant in disease types that form microaggregates, spheroids within a short duration of time, or for the investigation of organoid response to treatment. Particularly in patient-derived cultures, the ability to gain large amounts of information within a short window of opportunity, while the samples retain their tumour phenotype, is of paramount importance within an ex vivo pipeline.

Once the cell populations have been identified, the next step is to choose how to quantify a phenotypic response. In high-throughput assays which test many compounds in a single screen, there may need to be a compromise between accuracy and scale of detection. Drugs which target metabolic, proliferative or apoptotic pathways will show a direct phenotypic response in viability (see the next section). However, not all drugs are effectively assayed using viability. Drugs which target pathways associated with cell senescence, metabolism or which take a long time to take effect (for instance, causing genomic instability) may require assessment of specific biomarkers to predict drug efficacy. For immunotherapies, it is essential to retain, or re-introduce the immune complement, and then to assay the immune activity. For therapies which target angiogenesis, invasion and metastases, these drugs may only be effectively tested in a more complex ex vivo system, for instance using assembloids (described above).

### How to analyse ex vivo data?

Most ex vivo approaches require sophisticated analyses that can account for mixed populations of cells and combined treatments. How best to interpret ex vivo drug activity that translates accurately into clinical activity, remains an important question for ex vivo researchers. Currently reported measurable outputs from ex vivo approaches can be divided into single-output parameters, combined-output parameters, models which account for cell division, synergistic drug effects (listed in Table 3) and clinical outcomes (discussed in the ‘Future of ex vivo’ section). The most reported single output is percentage viability. This has been measured from a plethora of assays across both 2D and 3D platforms and analogous assays have been developed for PDE (Table 3). Indirect percentage-based observations are also reported, including relative cell fraction (Refs 19, 21), BP3 profiling (Ref. 118), sulphorhodamine B assay (Ref. 57), *z*-scores of caspase-3 activity (Refs 68, 119) and in PDX models, percentage tumour cell growth inhibition (TCGI) (Ref. 106) and motility contrast tomography (MCT, Onco4D™, NCT03164863).

**Table 3.** Example analysis techniques utilised in ex vivo screening

Single output read analysis	Example assay	Combined output read analysis
**Output**	**2D**	**Open source**
Percentage viability	IF apoptosis markers (Refs 24, 91, 120, 121)	DSS (v1, 2, 3, 4) (Ref. 122)
Percentage cells	FACS (Ref. 121)	PharmacoGx (Ref. 123)
Relative cell fraction (Refs 19, 21)	CTG (Refs 25, 43, 68, 119, 124, 125)	cellHTS (Ref. 126)
BP3 profiling (Ref. 118)	Summary of ex vivo models that have been utilised to direct patient treatment	HTS navigator (Ref. 127)
Caspase-3 activity (Refs 68, 119)	Nuclei counts (DAPI, Hoechst) (Refs 14, 128)	KNIME (Ref. 129)
Sulphorhodamine B assay (Ref. 57)		BREEZE (Ref. 130)
MCT (NCT0314863)	**3D**	
IC_50_ (Refs 14, 18)	PrestoBlue (Ref. 23)	**Commercial**
	TUNNEL (Ref. 98)	Dotmatics (Ref. 131)
	IHC (Refs 22, 98)	Genedata screener (Ref. 132)
	Apoptosis by morphology (Ref. 116)	
	TCGI (Ref. 106)	

CTG, CellTitre-Glo^®^; DAPI, 4′,6-diamidino-2-phenylindole; DSS, drug sensitivity score; FACS, flow cytometry; IC_50_, half-maximal inhibitory concentration; IF, immunofluorescence; IHC, immunohistochemistry; MCT, motility contrast tomography; MTT, 2,5-diphenyl-2*H*-tetrazolium bromide.

More robust methods of measuring drug response utilise multiple parameters from a drug response curve. The drug sensitivity score (DSS, versions 1, 2 and 3), developed by Yadav *et al*. combines multi-parametric dose–response information into a single metric, comparing drug response patterns between cancer and control cells, instead of reporting drug responses in cancer cells alone (Ref. 122). The DSS out-performed relative IC_50_ alone in scoring drug responses in vitro. This method is open access and available via a stand-alone R-package.

Traditional methods of measuring drug response (IC_50_, *E*_max_, AUC, etc.) are influenced by cellular replication. This is important to recognise in functional, or phenotypic drug sensitivity. The growth rate value is one metric which addresses this (Ref. 135), and has been adopted in a number of ex vivo studies which take screening results to clinical practice (Refs 19, 21, 26). Studying combinations of therapies requires specific analysis methods to account for synergic effects, which can be accounted using multiple methods including the Chou-Talalay or Mixlow method, or the Bayesian approach by Hennessey *et al*., all reviewed by Ma *et al*. (Ref. 136). However, we firmly believe analysis techniques will evolve rapidly over the coming decade, with the increase of artificial intelligence-based image analysis methods being utilise to assess morphology and cellular dynamics in response to treatment, as part of ex vivo approaches.

### How to report ex vivo results?

For the results of ex vivo approaches to benefit patients, the information has to be interpretable and intelligible to the clinical team managing patient treatment. Studies which have reported ex vivo determined treatment outcomes cite ‘review boards’ as the main avenue for reporting results back to the clinical team (Ref. 32). However, there is no published standardised methodology of how ex vivo reporting should be conducted. Parallels could be drawn from the UK Genomics England 100K project, which aimed to communicate relevant genetic results to clinicians to inform decisions about treatment and screening in germline variants. However, the consistency and utility of this reporting has had high geographical variation across the NHS. Ultimately, it is likely that the types of reports will reflect the health model and institution the ex vivo screening is being undertaken in (whether that be a public health service, such as the NHS, or insurance-based healthcare, whether the ex vivo screening is being undertaken at a local centre, or at a regional or national level), and whether ex vivo screening outputs are combined with the results of genomic testing to provide more integrated therapeutic recommendations. As such, a ‘one size fits all’ methodology for reporting ex vivo data to clinical teams may not be appropriate across such diverse geographic and institutional settings.

### Validation

The chosen ex vivo approach will need to be assessed across several factors to determine its effectiveness, including (1) linearity (which will dictate minimum sample requirement and tumour content), (2) criteria for variability (in both technical and biological replicates), (3) inter/intra-assay reproducibility, (4) image analysis reproducibility and (5) sample stability. Critical reagents, such as drug libraries used in the screen, drug plate manufacturer, stains, antibodies etc., will also require independent validation and testing prior to being used in a regulated setting. This is necessary for any diagnostic or predictive test, and together functions in defining pass/fail criteria. This is key in taking ex vivo screening methodology from a research background and applying it to patient stratification within a healthcare system.

## The future of ex vivo techniques

As this review has demonstrated, pan-cancer ex vivo techniques have a very rich research basis. However, ex vivo approaches in solid tumours have struggled to transition from a small-scale, predominantly research based technology to an integrated diagnostic test within healthcare settings. Here, we highlight details of clinical trials, both reported and those currently recruiting (Table 2), and the difficulties to overcome in demonstrating the clinical utility of ex vivo approaches to improve patients' cancer treatment.

Clinical trials utilising ex vivo approaches can be broadly distinguished into three types: translational validation, clinical validation and interventional, or treatment directing. Few of these clinical trials have published trial protocols, so instead information has been reviewed from clinicaltrials.gov. However, since this is not a peer-reviewed resource, it is likely that there will be discrepancies in the registered outcomes, compared with the published findings, once the studies have concluded.

There is some overlap between trials listed as ‘non-interventional’, but yet which do inform treatment decisions. In biliary malignancies, one study has incorporated a secondary outcome of ‘Physician-adjusted utility of profiling test results’ (NCT04561453). Investigators ask cancer-physicians whether the profiling results were helpful in the ultimate management of the patient in deciding adjuvant or palliative therapy, although they do not report collecting any prospective clinical data on patients who receive ex vivo-directed therapies. The trial is not considered interventional, the trial team report, since treatment with the ex vivo determined functional screen is not mandated in the trial protocol, but it is instead a clinician's decision whether they act on the results. Similarly designed studies, such as NCT03561207 in high-grade glioma and ovarian cancer, are more explicit about the prospective comparison between standard of care treatment responses from their ex vivo screen, and the clinical response of the patient. This study protocol does also allude to the inclusion of both FDA-approved and off-label FDA-approved therapies. This variation in outcome measurements is likely to have contributed to the slow translation of ex vivo techniques into clinical practice.

Although there is a wide literature base for ex vivo technologies, there are comparably few RCTs. The reason for this could be explained by any single, or combination of the aspects covered within this review: patient, disease and institutional factors, platform choice, variation between analysis techniques and lack of clinical information which challenge the transition from early phase, low-participant trials into larger, more expensive RCTs. Without evidence established through these more robust trials, it will not be possible to gain the support from public healthcare providers to invest in the necessary infrastructure and support service capacity. Although pharmaceutical companies are often eager to provide treatments for research capacity, it may be harder to convince them to provide drugs outside of their licenced indications in a systematic manner.

## Conclusion

This review highlights potential ex vivo approaches, which have, or could be used to predict patient response to treatment with chemotherapies and targeted inhibitors. Here we have presented the ex vivo techniques with the most clinical impact to date (patient-derived cultures, patient-derived cell lines), most automated and high-throughput capability (patient-derived primary cultures, patient-derived cell lines, organoids) and greatest biological relevance (organoids, assembloids, PDE and PDX models). All of these approaches have the potential to bridge the gap between identifying genetic targets, and patients benefiting from a directed therapy. All techniques have the potential of predicting patient response. However, based on the throughputs of therapeutics screened or turnaround time, some are better suited to drug development, such as PDX and PDE models. At the other end of the scale, patient-derived cultures and organoids have been used to direct patient treatment on a small scale and meet many of the logistical requirements to be transferred into a diagnostic environment.

Moving forward into a diagnostic environment ex vivo screening methodologies, such as patient-derived primary cultures and cell lines, could be readily integrated into regulated pathology laboratories, who are already part of the patients' tissue and result pathways and understand the regulatory requirement in healthcare systems. In addition, many steps in these methods, such as cell seeding, incubation, fixing, staining, microscopy and image analysis can all be heavily automated using liquid handlers, robotic transfer systems and bioinformatics pipelines, in a similar way to high-throughput NGS laboratories. Costs also need to be considered, with patient-derived primary cultures at the lower end of the scale and PDX models at the higher end. Additionally, the cost of these methods will increase significantly when being analysed in a more regulated diagnostic environment, compared with a research laboratory, with the addition of quality assurance, document audit trails and QC checks, and external regulation, which is another reason to integrate into existing diagnostic environment. Overall they promise great potential for biomarker and drug discovery, as well as delivering personalised medicine in both public and insurance-based healthcare models. Ex vivo approaches have the potential to add another functional dimension and provide actionable results to MDTs complementing genetics and histology in directing cancer patients' clinical treatment. Finally, we need large-scale observational or RCTs to evidence the potential benefits of ex vivo approaches before this could be integrated into healthcare models. Hopefully, some of the trials in Table 2 will accomplish this.
